# Bevacizumab-Induced Tumor Vasculature Normalization and Sequential Chemotherapy in Colorectal Cancer: An Interesting and Still Open Question

**DOI:** 10.3389/fonc.2021.751986

**Published:** 2021-09-24

**Authors:** Alessandro Ottaiano, Michele Caraglia

**Affiliations:** ^1^ Istituto Nazionale Tumori di Napoli, IRCCS “G. Pascale”, Naples, Italy; ^2^ Department of Precision Medicine, University “L. Vanvitelli” of Naples, Naples, Italy

**Keywords:** colorectal cancer, bevacizumab, oxaliplatin, normalization window, VEGF

## Introduction

**Figure 1:**
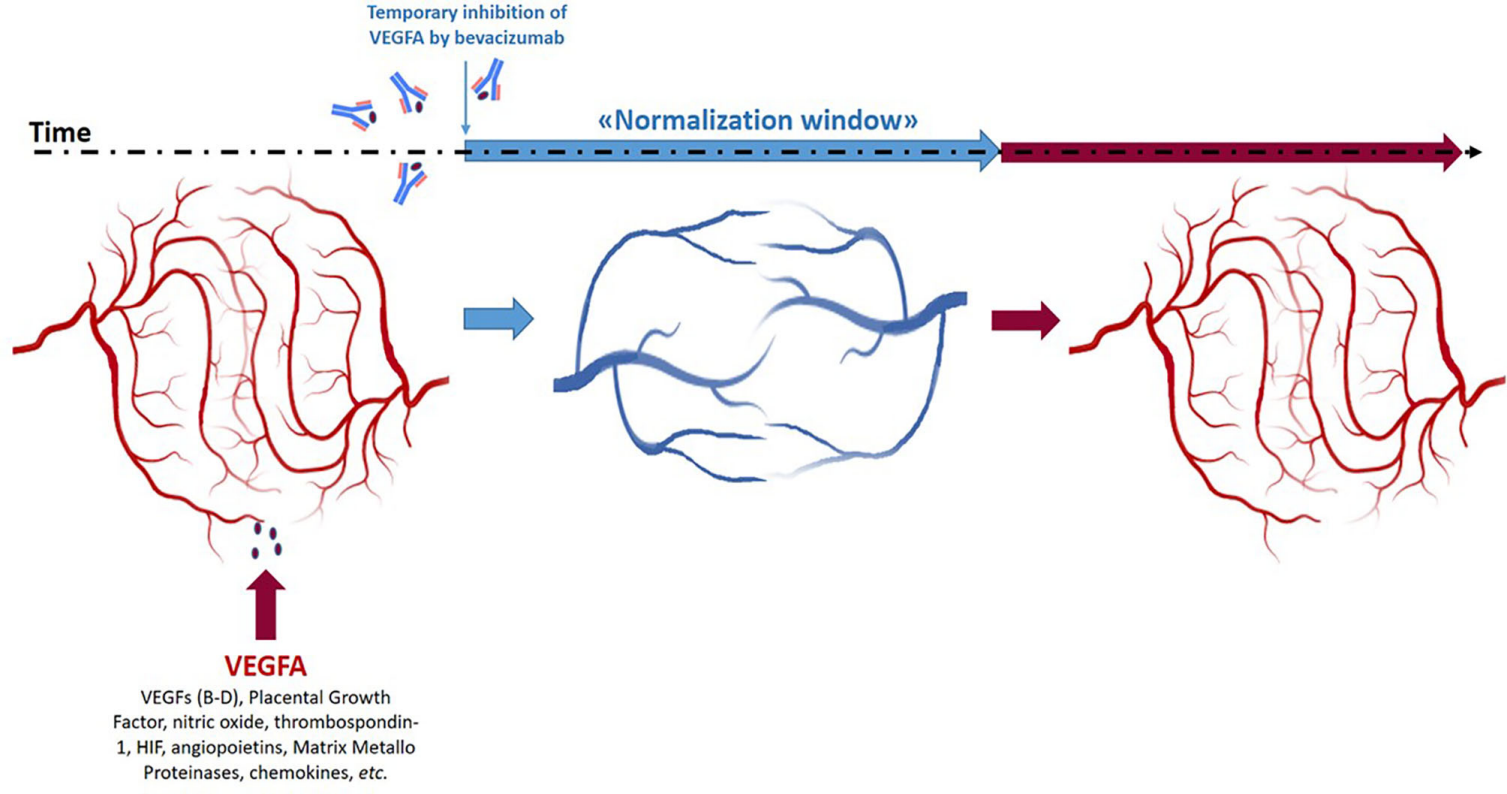
Schematic representation of the “normalization window” of anti-angiogenic treatment of tumor masses during time and space.

Bevacizumab is a recombinant fully humanized IgG1 targeting the Vascular Endothelial Growth Factor A (VEGF-A). The rationale of its use in oncology relies on the critical role of VEGF-A-induced neo-angiogenesis in the growth of many solid tumors (1–3). Since its discovery, many evidences suggested that it did not display direct antitumor action but rather contributes to improve the effects of associated chemotherapy. In fact, following bevacizumab administration, intratumoral vessels become morphologically more organized, and hypoxia and interstitial fluid pressure reduce (“vasculature normalization”); as a consequence, chemotherapy drug delivery into tumor masses ameliorates (4). Avallone et al. published the first randomized phase III study (OBELICS study) comparing the sequential administration of bevacizumab before standard oxaliplatin-based chemotherapy *versus* a traditional concomitant regimen in metastatic colorectal cancer (CRC) (5). The intent was to optimize bevacizumab and chemotherapy association by administering the chemotherapy in the “normalization window” and ameliorate the antitumor effects. The “normalization window” is the time window during which the anarchist texture of tumor vasculature becomes macroscopically more linear and less dense (1–4) (**Figure 1**). The authors fail to meet the primary end-point based on a difference in objective response rate (ORR) between the two arms. The odds ratio of response for experimental (ORR: 56.5%) *versus* standard arm (ORR: 57.4%) was 0.96 (95% CI: 0.55–1,68, P=0.89). We would add insights and prompt discussion making hypotheses on the causes of failure. This is important for scientific discussion and for planning future trials.

## The Heterogeneity of “Normalization Window” in Time and Space

The “normalization window” has an intrinsic heterogeneous nature in time and space. In fact, it is transient and closes as the anti-angiogenic drug effect cyclically reduces (**Figure 1**). A crucial question rises: which is the nadir of this effect? The theories prediction of this nadir should be integrated into study design to choose the best timing to administer chemotherapy. Unfortunately, this is still an unanswered question. Preclinical xenograft models with tumor cell lines are too homogeneous and far from the wild tumor human mechanisms. However, in some cases, the transient window is identified also earlier and later than 4 days timing used in the study by Avallone et al. (6). Most importantly, in clinical practice, with human subjects, the nadir of normalization is difficult to evaluate and is also physiologically heterogeneous (7–10). In a mathematical and intuitive modeling of the effect of anti-angiogenic drugs, Hutchinson et al. demonstrated that one of the most important parameters to identify the “normalization window” is the tumor volume rather than the number of metastatic sites accounted for a stratification factor in the randomization procedure of the OBELICS study. Hutchinson describes “normalization windows” ranging until 21 days (11).

## Discussion

Some considerations are raised that have not been considered by OBELICS investigators to interpret their results. Are 4 days sufficient to administer chemotherapy in a correct normalization window? The use of diffusion-weighted magnetic resonance (DWMR) imaging, which is widely accepted for monitoring intratumoral vasculature evolution, could have contributed to identify and “personalize” the normalization window. In fact, bevacizumab-induced tumor vasculature normalization should be evaluated and integrated into the design of future trials to adequately assess efficacy of sequential combination treatments (12). The authors’ choice to administer bevacizumab 4 days before chemotherapy was predominantly based on a previous study (BRANCH trial) (13) in rectal cancer; however, this is questionable since both clinical and biological behaviors of colon and rectal cancers, including their molecular signatures, are profoundly different (14). It has also to be considered that the usage of bevacizumab can induce modifications in the assets of cytogenetics and factors involved in the regulation of angiogenesis. In fact, although it was reported that bevacizumab-induced increase in VEGF did not affect the sensitivity of tumors to anticancer treatments, it cannot be excluded that other angiogenic factors can be modulated during single-agent bevacizumab administration inferring the treatment activity (15). Thus, a biomarker-based stratification of the patients (i.e., high *vs* low basal VEGF-A) should be required in order to evaluate the efficacy of the sequential administration. Moreover, it is reported that bevacizumab in CRC can determine profound metabolic changes peculiar of hypoxic conditions together with HIF (Hypoxia Inducible Factor) increased expression (16). This can, in turn, induce resistance to chemotherapy deserving HIF-blocking strategies to re-sensitize cancer to drugs (17).

In conclusion, we believe that the normalization effect of bevacizumab on tumor vasculature in CRC patients is a fascinating scientific question that remains to be determined as the optimization of the therapy through sequential administration with chemotherapy. In the future, we propose to integrate DWMR imaging in study design to adequately assess the “normalization window” and to perform biomarkers- and tumor volume-based stratification of patients for better interpretation of the results.

## Author Contributions

All authors listed have made a substantial, direct, and intellectual contribution to the work and approved it for publication.

## Funding

We receive funds from LILT (lega Italiana per la Lotta contro i Tumori) for open access publication fees.

## Conflict of Interest

The authors declare that the research was conducted in the absence of any commercial or financial relationships that could be construed as a potential conflict of interest.

## Publisher’s Note

All claims expressed in this article are solely those of the authors and do not necessarily represent those of their affiliated organizations, or those of the publisher, the editors and the reviewers. Any product that may be evaluated in this article, or claim that may be made by its manufacturer, is not guaranteed or endorsed by the publisher.
